# Prenatal Maternal Stress and the Risk of Asthma in Children

**DOI:** 10.3389/fped.2017.00202

**Published:** 2017-09-20

**Authors:** Konstantinos Douros, Maria Moustaki, Sophia Tsabouri, Anna Papadopoulou, Marios Papadopoulos, Kostas N. Priftis

**Affiliations:** ^1^3rd Department of Pediatrics, “Attikon” University General Hospital, School of Medicine, National and Kapodistrian University of Athens, Athens, Greece; ^2^Cystic Fibrosis Department, “Aghia Sophia” Children’s Hospital, Athens, Greece; ^3^Department of Paediatrics, School of Medicine, University of Ioannina, Ioannina, Greece

**Keywords:** maternal prenatal stress, offspring, wheezing, asthma, glucocorticoids, microbiota

## Abstract

Emerging evidence indicate that maternal prenatal stress (MPS) can result in a range of long-term adverse effects in the offspring. The underlying mechanism of MPS is not fully understood. However, its complexity is emphasized by the number of purportedly involved pathways namely, placental deregulated metabolism of maternal steroids, impaired maturation of fetal HPA axis, imbalanced efflux of commensal bacteria across the placenta, and skewed immune development toward Th2. Fetal programming probably exerts a pivotal role in the end result of the above pathways through the modulation of gene expression. In this review, we highlight the current knowledge from epidemiological and experimental studies regarding the effects of MPS on asthma development in the offspring.

## Introduction

About 40 years ago, Selye gave the first generic and comprehensive definition of stress as “the non-specific response of the body to any demand” (1). Stress represents the effects of any factor able to threaten the homeostasis of an organism; these either real or perceived threats are referred to as the “stressors” and comprise a long list of potentially adverse factors, which can be emotional or physical (2). Stressors provoke an integrated response from the central nervous system in an effort to restore or preserve homeostasis (3).

Extensive experimental animal studies and epidemiological observations allow us to surmise that events and conditions during intrauterine life can have a key role in many aspects of fetal development, affect birth outcomes, and what is more, influence subsequent child and adult health susceptibility for many common disorders with complex multifactorial etiology (4, 5). Maternal prenatal stress (MPS), in particular, may cause a series of alterations in the developing fetus, influence early life events, and have long-lasting consequences in the offspring (3, 5, 6). These alterations are mainly considered part of the fetal programming (or fetal imprinting) phenomenon. The general idea conveyed by these two synonymous terms is that there is a dynamic interplay between the genetic makeup and the environment; certain stimuli, acting during critical periods of fetal development, can trigger a series of adaptive mechanisms and reset gene expression, with profound and permanent consequences on the tissues’ structure and metabolic function. The molecular mechanisms underlying fetal programming are only partially unraveled, but what is clear is that the ensuing changes have long-lasting results in the function of affected tissues and organs, and can also pass from one generation to the next (7–9). Among the compounds with programming potentials are growth factors, cytokines, and hormones, all of which can be affected by stress (8).

The aim of this review is to summarize the increasing body of epidemiological and experimental evidence regarding the effects of MPS on asthma development in the offspring. The discussed human studies include a variety of stressors experienced during pregnancy such as, negative life events, anxiety, or depression. Similarly, animal studies have also been conducted with various types of stressors.

## Prenatal Maternal Stress and Offspring Outcomes

Maternal prenatal stress may result in a range of long-term consequences in the offspring (3, 10–12). Research in humans has demonstrated a relation between MPS and perinatal/postnatal outcomes. Our knowledge on either the immediate or the enduring effects of MPS in the offspring derives largely from animal studies (10); however, the amount of available information from research in humans has been accumulating rapidly over the past few years. The best-studied outcomes of fetal exposure to MPS are preterm birth and low birth weight (13–15). Indeed, a systematic review—39 peer-reviewed studies—strongly indicates that MPS increases the likelihood of preterm birth (15). Potential physiological pathways include behavioral, infectious, neuroinflammatory, and neuroendocrine mechanisms. Regarding low birth weight, it has also been noted that maternal psychological and social stress during pregnancy may affect human embryo and fetus development. This is believed to be the result of a dynamic interaction between inherited and acquired genetic alterations as well as environmental factors, particularly during intrauterine life (7). It has been demonstrated that levels of placental corticotrophin releasing hormone (CRH) are associated with the rate of fetal growth and body size at birth (16). MPS is also considered responsible for a variety of functional and morphological changes of the offspring’s brain, and a risk factor for conditions such as behavioral problems, learning disorders, high levels of anxiety, attention deficit hyperactivity disorder, autism, and schizophrenia (17–20). Furthermore, MPS has been associated with a higher risk for a variety of immune and metabolic alterations in the offspring such as asthma, allergic disorders, cardiovascular diseases, hypertension, hyperlipidemia, diabetes mellitus, and obesity (5, 7, 21–25).

Studies indicate that MPS may also affect the offspring’s bacterial colonization (26). Recent data support that non-pathogenic commensal bacteria are present in the placenta (26). This implies an efflux of commensal bacteria from mother to fetus through the placenta, and the establishment of an early fetal microbiota (27). Studies have shown that exposure to different prenatal factors may influence the richness and variety of the offspring’s gut microbiota, although clinically relevant consequences are yet to be determined (27–29). Experiments in animal models indicate that MPS can alter the offspring’s bacterial intestinal colonization (30).

## Stress in Pregnancy

Women are more vulnerable to stress during pregnancy due to extensive hormonal and physiologic changes. MPS can be realized—in a multidimensional approach—as comprised of stressful situations (stressors), perceptions or evaluations of these stressors (appraisals), and stress responses such as subjectively experienced emotions (31, 32). Associations between MPS, conceptualized at each of these three overlying stress levels, and child outcomes, have been reported (33).

There is only a small body of literature on the prevalence of MPS, but probably its influence on maternal health has been underestimated; available evidence indicates that prevalence of MPS is relatively high. In a cross-sectional study of 1,522 ethnically and economically diverse pregnant women receiving antenatal care at a university obstetric clinic in Seattle, WA, USA, MPS was recorded as being low to moderate in 78% and high in 6% of the participants. High levels of MPS were associated with depression, panic disorder, drug use, domestic violence, and suffering from two or more medical comorbidities (33). An earlier study, which used prospectively collected data from a cohort of 14,000 women, showed that the proportion of women with probable depression was 11.8 and 13.5% at 18 and 32 weeks of pregnancy, respectively (34). The most common causes of MPS seem to be anxiety and depressive symptoms, pregnancy-related anxieties, parenting stress, and work-related stress (35). During the prenatal period, the concomitant suffering from increased levels of anxiety and depressive symptoms has been shown to be the most important risk factor for adverse birth outcomes (35). It has to be mentioned here, that although anxiety and depression represent separate psychological entities, it is difficult to be clearly differentiated as they are strongly correlated with each other (36–38). The importance of MPS has been emphasized by the American College of Obstetricians and Gynecologists who recommend regular assessment of pregnant women for psychosocial risk factors and, if needed, supportive interventions to help women cope with the psychosocial stressors (36–38).

## Prenatal Maternal Stress and Immune Response—Airway Inflammation

There is very strong evidence, mainly from experimental studies, that MPS modulates the immune response of the offspring (3). It is therefore reasonable to hypothesize that MPS may be related to future disorders of the offspring, which develop through the dysregulation of innate and adaptive immune pathways, such as asthma (39).

The adaptive immune mechanism constitutes a critical pathway for the development of asthma, which is characterized by a Th2-dominated immune response (Il-4, Il-5, Il-13), and a down regulated Th-1 response (Il-2 and IFN-γ). Additionally, other types of T cell subsets, such as regulatory T cells, which produce Il-10 and TGF-β, as well as Th17 cells, which produce Il-17, may confer to asthma development possibly by skewing the immunological response toward the Th-2 type (40). It has been also recognized that innate immune mechanisms may also contribute to asthma development by leading toward an upregulated Th-2 response (39).

Veru et al. reviewed the animal studies that explored the relation between MPS and the offspring’s immune response (3). The review included only the studies that used exogenous/processive stressors which are considered to represent very closely the human psychosocial stress. The reviewed data indicated that MPS can lead to disequilibrium of the Th1/Th2 ratio, in favor of Th2 response. The cytokine production underlying this shift was affected by MPS exposure, though not to the same extent for each cytokine. MPS also affected, in a gender dependent way, the natural killer (NK) cytotoxicity and macrophages activity, which represent the functional activity of two essential components of the innate immunity. In general, the effect of MPS on the innate immunity function of the offspring is inhibitory, as MPS attenuates macrophages and neutrophils functions, such as spreading and phagocytosis, and decreases, at least in some models, NK cytotoxicity (41). MPS also modifies the response of T lymphocytes to mitogens and to specific antigens, but the type of modification is depended on the time of stressor application during pregnancy (41). This has been quite expected, as different periods of immune system vulnerability during gestation have been proposed on the basis of immunotoxicology data (41). These periods are depended on the maturation stage of immune system and they may be different among species.

Although there are several studies exploring the relationship of MPS and immune response of the offspring, there is only a small number of experimental studies investigating the effects of MPS on the airway inflammation in the offspring. Nogueira et al. (42) investigated the role of chronic mild MPS induced by continual changes in living and feeding conditions, on leukocyte infiltration in the airways of the rat offspring. They found that after sensitization to ovalbumin, the prenatally stressed offspring group exhibited a 50% increase of total leukocyte, eosinophil, and mononuclear cell count in the bronchoalveolar lavage (BAL), compared to the non-stressed offspring group. This difference reached statistical significance, whereas no such difference was seen in the neutrophil cell count in the BAL of the stressed and non-stressed group.

In another murine model (43), the effect of stress caused by exposure to a sound emitting rodent repellent device on pregnant mice, during the 12th and 14th days of gestation, was investigated. It was observed that airway hyper-responsiveness upon allergen aerosol challenge was significantly increased in the stressed female offspring group after sensitization to ovalbumin. A relative increase of eosinophil cell count in the BAL was also observed, indicating increased susceptibility toward airway inflammation. It should be mentioned however, that exposure to a single stressor on the 12th day of gestation, was not sufficient to induce a tendency toward airway hyper-reactivity. This observation is in line with the hypothesis that the development of immune system during gestation is characterized by windows of vulnerability. In this animal model, the ratios of Th2/Th1 cytokines in the blood and BAL of the offspring were also assessed, and the results showed a domination of Th2 cytokines in the blood. A Th2 cytokine profile was also induced in naive T cells by lung dendritic cells from prenatally stressed offspring. It was also observed that total serum IgE was significantly elevated in the stressed sensitized group, implying a degree of dysregulation of the humoral immune response.

A major mediator of the MPS action on the offspring’s immune response is maternal glucocorticoids. The existing evidence supports that a fraction of the maternal glucocorticoids cross the placenta, deregulates the hypothalamic–pituitary–adrenocortical (HPA) axis of the fetus, and augments the fetal glucocorticoid production (41). In turn, elevated glucocorticoids of the fetus may lead to a predominance of Th-2 response (44). It is also known that epigenetic pathways contribute to the regulation of immune development (45). MPS may epigenetically modify key immune genes, leading to an altered immune response of the offspring. Oxidative stress (46) as well as maternal catecholamines and opioids (41) may also play the role of mediator.

It seems, therefore, that there are multiple candidate mediators of the MPS effects on the offspring’s immune development. However, irrespective of the type of mediator, the main deviation of the immune response in the prenatally stressed offspring is the up-regulation of Th-2 response which may predispose in asthma development.

## Pathways through Which MPS may Affect Offspring Outcomes

**Figure 1:**
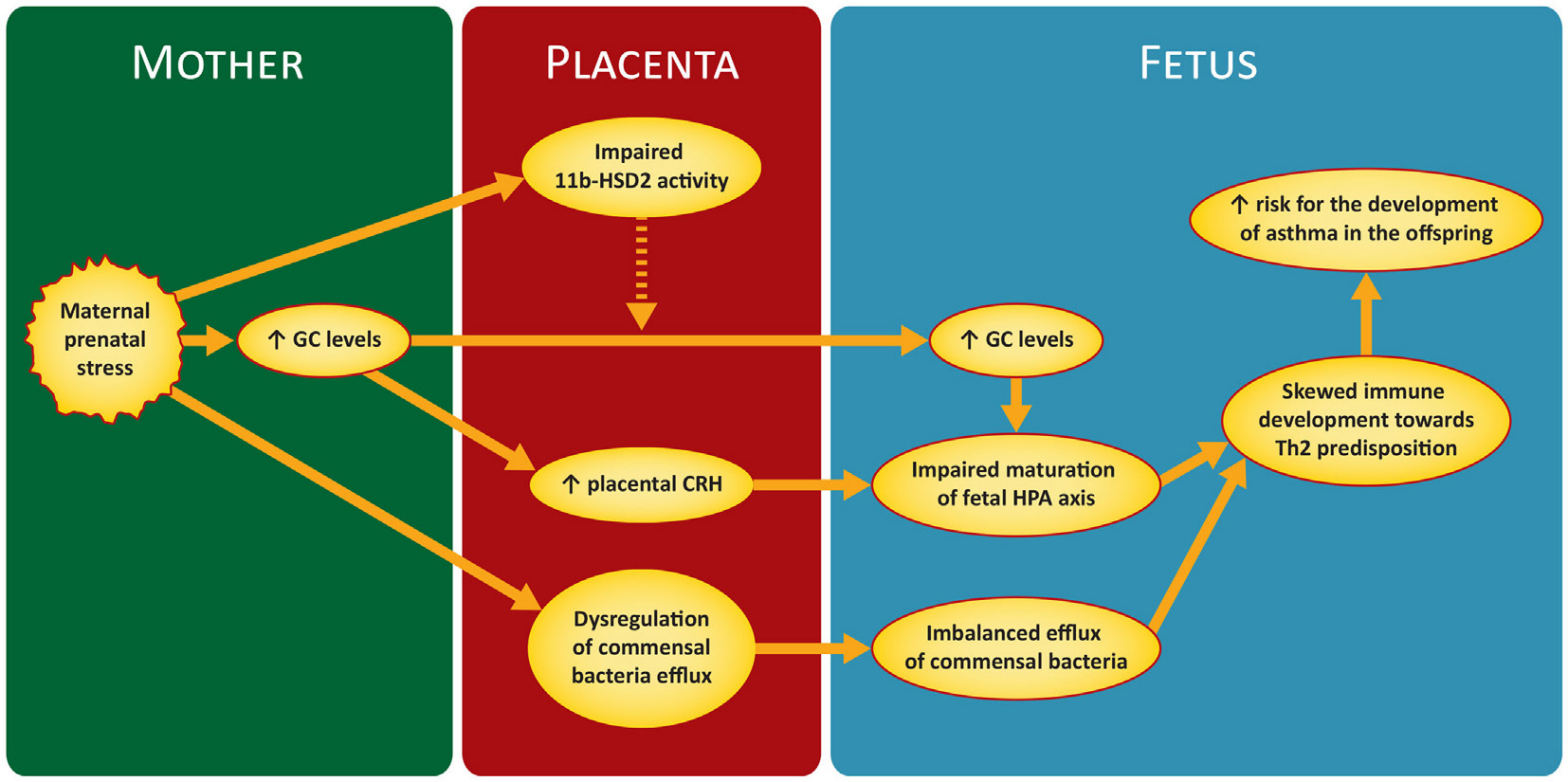
Proposed mechanism of maternal prenatal stress in asthma predisposition in the offspring. GC, glucocorticoids; CRH, corticotrophin releasing hormone; 11 b-HSD2, 11-b hydroxysteroid dehydrogenase-type 2; HPA, hypothalamic–pituitary–adrenal.

Figure 1 illustrates briefly the mechanisms discussed in the next sections.

### Maternal Environment

The most important mechanism involved in the association between MPS and offspring outcomes is the hypothalamic–pituitary–adrenal (HPA). When a mother is exposed to a stressor, her HPA axis is activated and releases a number of hormones. The most important of them is cortisol; due to their highly lipophilic nature, GC can theoretically pass easily through the placental barrier and reach the fetus. In essence, however, the placenta regulates dynamically the amount of GC that will finally reach fetal circulation (47). Indeed, only a small percentage of the GC reaching placenta during periods of MPS will manage to pass into fetal circulation (approximately 10–20%) (48). Nevertheless, because of the low normal levels of fetal GC, the transfer of even this small amount can increase significantly GC concentration in the fetal circulation, suppress the fetal HPA axis, and have an impact on its normal maturation (49). Furthermore, it seems that increased maternal cortisol levels can stimulate the production of placental CRH with resultant stimulation of the fetal HPA axis (49, 50) and further increase of fetal cortisol. Glucocorticoid receptors (GRs) are ubiquitous throughout nucleated cells and so, if there is an increase in GC levels in fetal circulation, gene expression and development of many organs and tissues will probably be affected. Fetal lung maturation is known to be one of the organs highly affected by high GC concentrations (50, 51). Nevertheless, it has to be emphasized, that GC are not able on their own to fully explain the whole range of fetus and offspring impairment from MPS. Maturation of the fetal HPA and GR expression begin during late pregnancy (52–54). As a consequence, vulnerability of the fetus to excessive maternal GG before this period is limited.

In an experimental animal study (55), it was demonstrated that the offspring of mice mothers stressed during pregnancy developed airway hyperactivity after sensitization with ovalbumin. Stressed pregnant mothers had significantly elevated corticosterone levels compared with non-stressed controls. Authors further showed that the administration of exogenous dexamethasone to pregnant mice led to offspring with asthma vulnerability. On the other hand, administration of metyrapone, which blockaded stress induced GC, abolished the asthma predisposition of the offspring. The results of this study provide experimental support on the role of MPS-related GC toward asthma predisposition in the offspring.

### Placenta

Maternal prenatal stress reduces uteroplacental blood flow by trigerring a fetal stress response through fetal HPA axis activation (56). But besides that, the role of placenta in modulating and moderating fetal reactions and programming, in response to MPS, is of pivotal importance. Placenta’s protective function from MPS is mainly employed by the enzyme 11-β hydroxysteroid dehydrogenase-type 2 (11β-HSD2). The enzyme’s role is to attenuate the effects of excessive maternal GC by converting cortisol into cortisone, which is a much less active metabolite (57). Unfortunately, despite the unequivocal protection from the surge of maternal GC during MPS, 11β-HSD2 is far from being the ideal buffering mechanism. The levels of 11β-HSD2 increase with the progression of gestation, but fall abruptly near term (58, 59) when, as it has been aforementioned, fetus is more vulnerable to the programming potential of maternal GC. Furthermore, it has been shown, both in humans and animal models, that MPS can reduce by itself the expression and activity of 11β-HSD2 (59–61).

The protective effects of 11β-HSD2 seem to be related only with the effects of acute stress. Exposure to chronic stress can have detrimental effects on the 11β-HSD2-based protection mechanism. In an animal model, it was demonstrated that exposure to acute stressors resulted in prompt up-regulation of 11β-HSD2, whereas, in cases of chronic MPS the ability for upregulation of the enzyme’s activity in response to an acute stressor was reduced by about 90% (62). Other studies have demonstrated that chronic MPS can downregulate 11β-HSD2, increasing this way the amount of cortisol that crosses the placenta (63). This downregulation of 11β-HSD2, is possibly attained through DNA methylation (60).

Concisely, 11β-HSD2 is not directly connected with the mechanisms involved in the development of asthma propensity in the offspring. However, it is probably indirectly involved since its role is to provide fetus with a protective barrier from the surge of maternal GC during MPS.

### Catecholamines

Sympathetic–adrenal–medullary (SAM) system, with its response hormones adrenaline and noradrenaline, plays a central role in stress. The secretion of these hormones comes as an immediate reaction to stress, contrary to HPA that releases its effector hormones more gradually (64). Despite SAM system being an integral part of stress reactions, catecholamines have attracted limited attention regarding their role in MPS and fetal consequences. This is probably due to the fact that catecholamines are hydrophilic and apparently not able to cross the placenta barrier in physiologically significant concentrations (65). Apart from that, the vast amount of catecholamines that reach the placenta are metabolized to inactive forms through the enzymes monoamine oxidase and catechol-O-methyltransferase (66).

In studies conducted in human placental tissue and animals, it was shown that only a minor quantity of catecholamines was able to be transferred from mother to fetus (67–69). Some older evidence, however, suggests that catecholamines are able to indirectly influence fetal metabolism by affecting the uteroplacental perfusion. In an animal study, high levels of circulating maternal catecholamines led to constriction of placental blood vessels, reduction in glucose supply to the fetus, and activation of fetal catecholamine release (70). However, the evidence implying an association between MPS and changes in fetal circulation are inconclusive, and even if this relation does exist it is transient in nature and limited to the temporary activation of the SAM system, after an acute stressful situation (65). It is clear from the above that there is paucity of data to support the implication of catecholamines in the development of asthma predisposition in the offspring and any role ascribed to SAM should be considered strictly hypothetical.

### Gut Microbiota Alterations

Another conceivable indirect mechanism through which MPS may also affect the fetus, is the formation of neonate’s gut microbiota. The significance of this, not yet fully confirmed mechanism, can be realized with the developmental and regulatory role of microbiota in many aspects of human physiology, including the immune system, the central nervous function, and the HPA (71–73). It seems that from the first days of life, a bidirectional communication system—the so-called “gut–brain” axis—is established between gut microbiota and the above systems; molecules such as cytokines, neuromodulators, and neurotransmitters act as messengers for this interaction (71, 73–75).

Until quite recently, the conventional belief was that fetus and its intrauterine environment remained sterile till the time of delivery (76). Although it is true that the degree of microbial colonization during and immediately after delivery is so enormous that within days the bacteria are by far more numerous than the newborn’s own cells, recent studies have demonstrated that a small, though not insignificant, number of bacteria is present in the intrauterine environment. This finding implies that the foundations of human microbiota lay in the prenatally occurring bacterial efflux from mother to fetus. Bacteria most likely escape into circulation from maternal intestine, and through the blood stream, enter the amniotic fluid. Fetus swallows the amniotic fluid providing this way the fetal intestine with its first bacterial load (32).

A neonate’s gut should be colonized by a diverse and balanced microbial population in order to establish a healthy microbiota (77). MPS disrupts the balance of maternal intestinal microbiota and creates a disequilibrium in the bacterial efflux to fetus and offspring (32). It has to be stressed, however, that much of the abovementioned remain largely unproved, and the development of fetal microbiota, as well as its significance, are still not entirely clear (78).

Experimental animal studies have shown that MPS alters the offspring’s bacterial intestinal colonization (30, 79, 80). Recently, Jasarevic et al. (81) showed that MPS changes vaginal microbiota and this alteration is transmitted at birth to the offspring’s gut microbiota. They also showed that MPS affects offspring’s microbiota in a temporal and sex specific manner (82). In a clinical study of 56 women–infants pairs (83), it was found that MPS was one of the determinants of the offspring’s microbiota composition. This effect reached its peak at 80 days, and it was still detectable at 110 days. High levels of MPS were associated with larger than usual numbers of Proteobacterial groups, and a relative dearth of lactic acid bacteria.

As far it regards the relation between MPS, microbiota, and asthma pathogenesis, the abovementioned findings, interpretations, and assumptions, could be incorporated in a proposed model with two interlaced pathways. In the first one, the imbalance of an infant’s intestinal microbiota may deviate the development of systemic immune toward a more “asthma prone” Th2 direction. The second one lies within the concept of “Common Mucosal Immune System” (84) and suggests a “crosstalk” between mucosal compartments from different organs; microbiota driven modulations in mucosal immunity at one site, may be transferred to a mucosal surface of another site. The above proposed model can be considered as part of the more general context of hygiene hypothesis (85).

## The Association with Childhood Asthma

It is more than 15 years that evidence from cohort studies and animal models linked, increasingly stronger, MPS with the development of childhood asthma.

Observations from epidemiological studies challenged researchers to investigate the hypothesis, eloquently described by Barker (86–88), of the antenatal origin of many adult diseases. The field of MPS was conveniently available for the studies that followed to test this hypothesis. Among other subjects, researchers focused on the prenatal origin of asthma and allergies in children. Following studies in animal models that linked physical or psychological stress with allergy and asthma in the first decade of the 21st century, researchers worked on the early-life stress and the consequences in asthma development later on.

In 2006, a team from Charité, Berlin, Germany, described an increased vulnerability toward asthma specific clinical features in prenatally stressed offspring mice (43). The same year in Japan, researchers presented the long-term asthmogenic effects of early life psychological and physical stress in mice (89). Eight years later, Lim et al. (55) exposed mice on the 15th day of pregnancy, to an 1-h restraint stressor and assessed offspring for the development of asthma susceptibility. Increased asthma susceptibility was found only in those offspring born from stressed mothers. It appears that inflammation-related hormones during pregnancy may represent a common pathway, shared by various different, associated or not with stress, factors through which the offspring’s susceptibility to develop asthma is affected.

During the same period, in the past 15 years, interesting data resulted from epidemiological studies.

Wright et al (90) conducted a prospective cohort study and reported a positive association between caregiver perceived stress by regular telephone interview and risk for wheezing episodes in children younger than 14 months of age. Few years later, a large cohort study in Canada, showed that maternal stress in early childhood, is positively associated with asthma at the age of 7 years (91). Authors used health care data in order to identify stress which was defined as “a combination of depression and anxiety” (91). The authors also found that the prevalence of asthma was dose related with the duration of exposure to stress. This interesting information was confirmed from another birth cohort, the Avon Longitudinal Study of Parents and Children (92), that assessed and scored the anxiety in pregnant women at 18 and 32 weeks of gestation and the occurrence of asthma in their offspring at school-age. The likelihood of asthma at age 7.6 years was higher in children whose mothers had been classified (at 32 weeks of gestation) in the highest anxiety scores compared with the corresponding mothers with anxiety scores in the lowest quartiles. Additionally, after adjusting for confounders such as postnatal anxiety, they showed that there was a dose–response relationship. More recently, a group working in Canada reported their observations on 68 mothers having experienced a natural disaster during their pregnancy. They noted that the results of early-life events associated with psychological distress, on the development of allergic airway inflammation in later-life depend on both the quality and intensity of the early-life stress stimuli (93).

In the Western Australian Rhine Study, researchers looked at the association of prenatal adverse life events during pregnancy with asthma and allergy of offspring followed until the age of 14 (94). They found that prenatal adverse life events increased the likelihood for atopic diseases, which was enhanced in the absence of a maternal atopic predisposition. The risk of asthma and/or eczema was positively correlated with the number of negative life events during the second half of pregnancy. The results were more pronounced for asthma than allergic rhinitis. Similar results were reported from Sweden and Italy with children exposed to stressful life events during pregnancy (95, 96).

In a prospective cohort from the Netherlands (97) researchers assessed the associations of MPS with offspring wheezing until the age of 6 years. The study included 4,848 children and showed that psychological distress in pregnancy was associated with significantly higher risk of wheezing. The results were independent from paternal psychological distress during pregnancy or maternal and paternal psychological distress after delivery.

The relationship between MPS and asthma in the offspring seems to be influenced by the type of MPS and the age of onset of asthma symptoms. In a large Danish cohort (98), prenatal stress was retrospectively assessed using as indicator the bereavement due to the death of a close relative 12 months prior to or during pregnancy. A significant association was observed between MPS and the risk of asthma before the age of 3 years. This association, however, was not found in children 4–15 years old, unless their mothers had lost a child prior to pregnancy. In another Danish cohort (99) that included 32,271 pregnancies, an association was found between MPS and the risk of asthma and atopic dermatitis at the age of 7 years. In the latter cohort, the surrogate for MPS was the psychosocial job strain.

Strong results have arisen from a recent meta-analysis that included 10 relevant studies. Overall, the study showed that the prevalence of wheezing or asthma was higher in children whose mothers had been exposed to some kind of prenatal stress than in mothers who had not experienced such a distress (100).

## Conclusion and Future Perspectives

Several types of MPS seem to increase the vulnerability of the offspring to asthma through interrelated pathways. Maternal GC, along with the improper activation of placental–fetal HPA axis, are considered the main mediators of this predisposition. However, there are various other pathways through which maternal stress may increase the offspring’s propensity for asthma. Amongst them, MPS imposed imbalances in fetal and offspring’s microbiota, is the less explored and the more promising to unravel—at least partially—the complex association between MPS and asthma. There is need for further studies with subjective and objective measures of stress, as well as objective measures of asthma, in order to elucidate the underlying signaling pathways, understand how they interact with each other, and draw concrete and meaningful clinical inferences. The natural next step—and maybe a stepping stone toward the medical communities’ efforts aiming at the prevention of asthma—would be the design and development of mother-centered interventions during pregnancy focusing on how to cope with particular stressors. This may prove to be an efficient way to prevent asthma—and maybe other ailments—predisposition in the offspring.

## Author Contributions

KD designed the article and wrote Introduction, Stress in Pregnancy, Maternal Environment, and Placenta sections. MM wrote Prenatal Maternal Stress and Immune Response—Airway Inflammation section. ST wrote Prenatal Maternal Stress and Offspring Outcomes section. AP wrote Catecholamines and Placenta sections. MP wrote The Association with Childhood Asthma section. KP equally designed the article, read, and made comments on the manuscript.

## Conflict of Interest Statement

The authors declare that the research was conducted in the absence of any commercial or financial relationships that could be construed as a potential conflict of interest.
